# Understanding the potentiality of accelerator based-boron neutron capture therapy for osteosarcoma: dosimetry assessment based on the reported clinical experience

**DOI:** 10.1186/s13014-017-0860-6

**Published:** 2017-08-15

**Authors:** Silva Bortolussi, Ian Postuma, Nicoletta Protti, Lucas Provenzano, Cinzia Ferrari, Laura Cansolino, Paolo Dionigi, Olimpio Galasso, Giorgio Gasparini, Saverio Altieri, Shin-Ichi Miyatake, Sara J. González

**Affiliations:** 1 Department of Physics, University of Pavia, via A. Bassi 6, 27100 Pavia, Italy; 2 National Institute of Nuclear Physics (INFN), Unit of Pavia, via Bassi 6, 27100 Pavia, Italy; 3 National Atomic Energy Commission (CNEA), Av. General Paz, 1499 Buenos Aires, Argentina; 4 National Scientific and Technical Research Council (CONICET), Av. Godoy Cruz, 2290 Buenos Aires, Argentina; 5 Department of Clinic-Surgical Sciences, Experimental Surgery Laboratory, University of Pavia, via Ferrata 9, 27100 Pavia, Italy; 6 Polyclinic S. Matteo Foundation, Viale Golgi 19, 27100 Pavia, Italy; 7 Department of Orthopaedic and Trauma Ortopaedic Surgery, University of Catanzaro, Catanzaro, Italy; 8 Cancer Center, Osaka Medical College, Takatsuki, Japan

**Keywords:** Boron neutron capture therapy, Osteosarcoma, Accelerator-based BNCT, Photon iso-effective dose, Mixed-field dosimetry

## Abstract

**Background:**

Osteosarcoma is the most frequent primary malignant bone tumour, and its incidence is higher in children and adolescents, for whom it represents more than 10% of solid cancers. Despite the introduction of adjuvant and neo-adjuvant chemotherapy that markedly increased the success rate in the treatment, aggressive surgery is still needed and a considerable percentage of patients do not survive due to recurrences or early metastases. Boron Neutron Capture Therapy (BNCT), an experimental radiotherapy, was investigated as a treatment that could allow a less aggressive surgery by killing infiltrated tumour cells in the surrounding healthy tissues. BNCT requires an intense neutron beam to ensure irradiation times of the order of 1 h. In Italy, a Radio Frequency Quadrupole (RFQ) proton accelerator has been designed and constructed for BNCT, and a suitable neutron spectrum was tailored by means of Monte Carlo calculations. This paper explores the feasibility of BNCT to treat osteosarcoma using this neutron source based on accelerator.

**Methods:**

The therapeutic efficacy of BNCT was analysed evaluating the dose distribution obtained in a clinical case of femur osteosarcoma. Mixed field dosimetry was assessed with two different formalisms whose parameters were specifically derived from radiobiological experiments involving in vitro UMR-106 osteosarcoma cell survival assays and boron concentration assessments in an animal model of osteosarcoma. A clinical case of skull osteosarcoma treated with BNCT in Japan was re-evaluated from the point of view of dose calculation and used as a reference for comparison.

**Results:**

The results in the case of femur osteosarcoma show that the RFQ beam would ensure a suitable tumour dose painting in a total irradiation time of less than an hour. Comparing the dosimetry between the analysed case and the treated patient in Japan it turns out that doses obtained in the femur tumour are at least as good as the ones delivered in the skull osteosarcoma. The same is concluded when the comparison is carried out taking into account osteosarcoma irradiations with photon radiation therapy.

**Conclusions:**

The possibility to apply BNCT to osteosarcoma would allow a multimodal treatment consisting in neo-adjuvant chemotherapy, high-LET selective radiation treatment and a more conservative surgery.

## Introduction

Osteosarcoma is the most frequent primary malignant bone tumour, and its incidence is higher in children and adolescents, for whom it represents more than 10% of solid cancers [1]. Currently, the standard treatment of this malignancy consists in preoperative (neo-adjuvant) chemotherapy followed by surgical removal of all detectable disease (including metastases, that are yet present in 80% of patients at diagnosis), and postoperative (adjuvant) chemotherapy [2]. The standard route of neo adjuvant chemotherapy, surgery and adjuvant chemotherapy is effective in approximately 70% of the patients with localized disease, but for patients with metastases, the long term survival rate is lower than 20% [3]. Neo-adjuvant chemotherapy allows surgery to achieve wide or radical margins while adjuvant chemotherapy is essential to control micro-metastases (responsible for distant metastases and local recurrences) [4, 5]. In the last decades, the objective of avoiding limb amputation and preserving the functional and cosmetic status of patients without decreasing the survival rate has gained importance, leading to surgical techniques named limb-salvage, nowadays performed in 80% of the patients [6].

Osteosarcoma has been generally considered as a radio-resistant tumour, however it has been suggested that a large single fraction dose of radiotherapy may be effective. Radiotherapy has been used in small series of patients, and has been reported as an option for local treatment of unresectable tumours, or as palliation. Given the difficulty to deliver high photon doses in single fraction due to the tolerance of surrounding tissues, patients have been recently treated with charged particles (protons and carbon ions), that allow a very conformal dose distribution. Some other patients have received intraoperative radiotherapy and even extracorporeal irradiation [7–9].

**Fig. 1:**
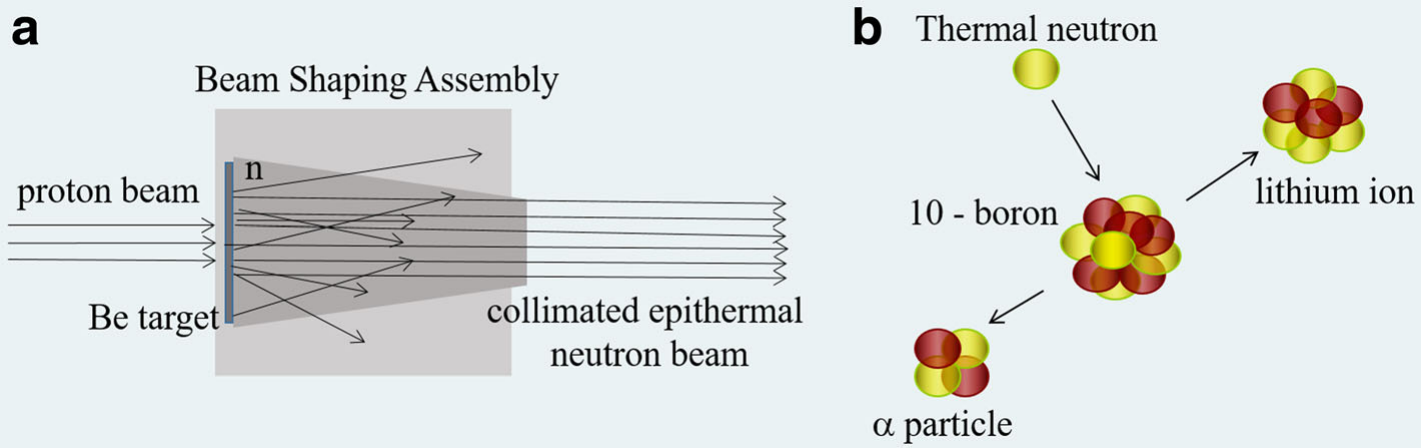
**a** Sketch of the neutron beam production by accelerator: protons interact in the target via (p,n) reaction on beryllium, neutrons are moderated and collimated in the Beam Shaping Assembly (BSA). **b** scheme of charge particle production by thermal neutron capture in ^10^B

Boron Neutron Capture Therapy (BNCT) is an experimental form of radiotherapy based on the irradiation of a tumour with low energy neutrons after the target enrichment with atoms of 10-boron (^10^B) [10]. Low energy neutrons are captured by ^10^B with high probability, generating two high-Linear Energy Transfer (LET), short range particles that release all their energy in a distance comparable to a cell diameter (Fig. 1b). In this path, the charged particles cause non reparable damages to the DNA, thus killing the cell. If ^10^B is taken up preferentially in the tumour, the neutron irradiation can deliver a therapeutic dose, without affecting the surrounding healthy tissues. The most interesting characteristic of this treatment is its selectivity, that depends on the bio-distribution of ^10^B, rather than on the irradiation beam. Potentially, it can be effective also for disseminated metastases or for isolated tumour cells surrounded by normal tissues. The drug currently used in clinical practice to carry ^10^B into tumour is Boronophenylalanine (BPA) [11].

Up to now BNCT has been applied in many centres in the world provided with research nuclear reactors, the only neutron source intense enough to produce neutron beams for the therapy, and clinical trials have been dedicated to brain tumours, head and neck recurrent and primary tumours, skin melanoma, lung cancer [10, 12, 13]. Today, technology is ready to produce neutron beams from high-current proton accelerators coupled with Li or Be targets, and the possibility to install these machines in hospitals makes BNCT a more widespread option. Two BNCT clinical facilities based on accelerators technology are already operating at Kyoto University Research Reactor Institute (KURRI) and at Southern Tohoku BNCT Research Centre in Japan [14].

Italian National Institute of Nuclear Physics (INFN) designed and constructed a Radio Frequency Quadrupole (RFQ) proton accelerator, meant to be used as a neutron source for BNCT clinical applications [15]. The RFQ delivers 30 mA of 5 MeV protons. It is coupled to a beryllium target, and has a neutron yield of about 10^14^ s^−1^ with maximum energy around 3.2 MeV. A Beam Shaping Assembly (BSA) is being designed in order to obtain an epithermal neutron beam peaked between 1 and 10 keV, energy range that was proven to be the most indicated for an advantageous dose distribution for deep-seated tumours like lung cancer [16]. The BSA design is based on the physical properties of the beam and it is optimized according to the dose distributions obtained in realistic clinical cases by treatment planning simulations (Fig. 1a).

Dosimetry in particle therapy requires the use of a formalism to express the dose in terms of photon conventional radiotherapy.

BNCT is characterized by a mixed radiation field composed by: high LET alpha and lithium ions from thermal neutron capture by ^10^B, intermediate LET protons from thermal neutron capture by ^14^N and neutron elastic collision with H nuclei, and low LET gamma-ray from the thermal neutron capture reaction by H and the gamma radiation in the irradiation beam. The energy released by the high and intermediate LET particles causes a higher severity of biological damage than photons.

Classically, BNCT dose has been reported as *equivalent dose* (in Gy-Eq), obtained by multiplying each dose component by Biological Effectiveness/Compound Biological Effectiveness (RBE/CBE) factors derived from cell survival curves, for a fixed end point [17]. González and Santa Cruz [18] showed that BNCT outcome as a function of the dose delivered to melanoma nodules, is hardly comparable with photon therapy clinical outcome, the RBE-weighted doses being artificially high. Their new approach of *photon iso-effective dose* (in Gy (IsoE)) has demonstrated to be more coherent in terms of dose-response outcome. In this work, both the fixed weighting factors and the iso-effective dose have been applied to calculate dose distribution in a representative case of parosteal osteosarcoma of the distal femur.

BNCT has been clinically applied for the first time in Japan in two cases: a recurrent maxilliary sarcoma (fibroblastic osteosarcoma) [19] and a skull radio-induced osteosarcoma [20]. The latter treatment ensured a stable local control of the tumour that did not show any recurrence for 17 months, and the overall status of the patient markedly improved already few days after the treatment. This clinical case was used as a reference for the dosimetry calculations in the representative case of parosteal osteosarcoma of the distal femur, and to draw conclusions about the feasibility of this therapeutic approach.

## Materials and methods

### Osteosarcoma cell survival

The survival of UMR-106 rat osteosarcoma cell line was determined as a function of the dose, for three cases: neutron irradiation after BPA treatment (BPA-BNCT), neutron irradiation alone (beam-only) and photon irradiation. The experimental points were fitted using a modified linear quadratic model, that provided both CBE/RBE factors for osteosarcoma, and the parameters for the iso-effective dose calculation. The assessment of cell survival as a function of the dose has been previously reported [21]. Neutron irradiations took place in the Thermal Column of the TRIGA reactor, University of Pavia, and photon irradiation (Co-60 source) at the S. Matteo Polyclinic Foundation in Pavia. Boron concentration in cells was measured for each experiment in samples treated the same day and undergoing the same procedure as the ones irradiated for survival assessment. Boron naturally present in cells was also measured and included in the dose calculation for the beam-only component. All measurements were carried out by neutron autoradiography [22].

The radiation dose absorbed by cells has been calculated with MCNP6 [23], without approximation of charged particles equilibrium. T-75 flasks with adherent cells (described as a uniform layer of soft tissue) and 20 ml of culture medium were simulated in the irradiation position. Gamma from background and gamma generated by (n,gamma) reactions in H were transported in a gamma-electron coupled mode, and the dose due to energy deposition of electrons was calculated in the cell layer. Alpha particles, lithium ions and protons due to neutron capture in ^10^B and in ^14^N were generated uniformly in the cell layer and transported. The results were normalized by the rate of capture reactions calculated in a previous simulation of the whole reactor with the flasks in the irradiation position.

### Boron concentration in tissues

Boron concentration in tumour and muscle to be used in the treatment planning comes from preliminary measurements performed with a rat model of osteosarcoma [24] treated with BPA, 300 mg/Kg b.w. The pilot study consisted in animals treated with both intra-peritoneal and direct BPA injection in the limb. However, the latter administration route was proven less efficient in enriching tumour with respect to normal tissues, thus the results reported in this work correspond to intra-peritoneal injection.

The protocol involving animal model was approved by the Ethical Committee for animal experiments, Department of Internal Medicine and Medical Therapy, University of Pavia (reference nr 1/2012).

Measurements were carried out by alpha spectrometry [25] and quantitative neutron autoradiography [22] and imaging of ^10^B distribution in this tissue sections was obtained by neutron autoradiography [26]. A concentration equal to the one of normal muscle was assumed in healthy bone, based on preliminary results of bio-distribution studies carried out in sheep treated with the same dose of BPA in Argentina [27]. Concentration in skin was set 1.5 times the concentration in healthy muscle as assumed in the clinical trials of melanoma [12, 28].

### Treatment planning simulations

The clinical case analysed in this work is a male patient, 17 years old, showing a parosteal osteosarcoma arising from the posterior aspect of the distal metaphysis of the femur and extending to the popliteal fossa (tumour volume about 50 cm^3^). On the MRI imaging (Fig. 1) the mass was quite well-delimited and the appearance of the new-formed tissue had both blastic and lytic features.

**Fig. 2:**
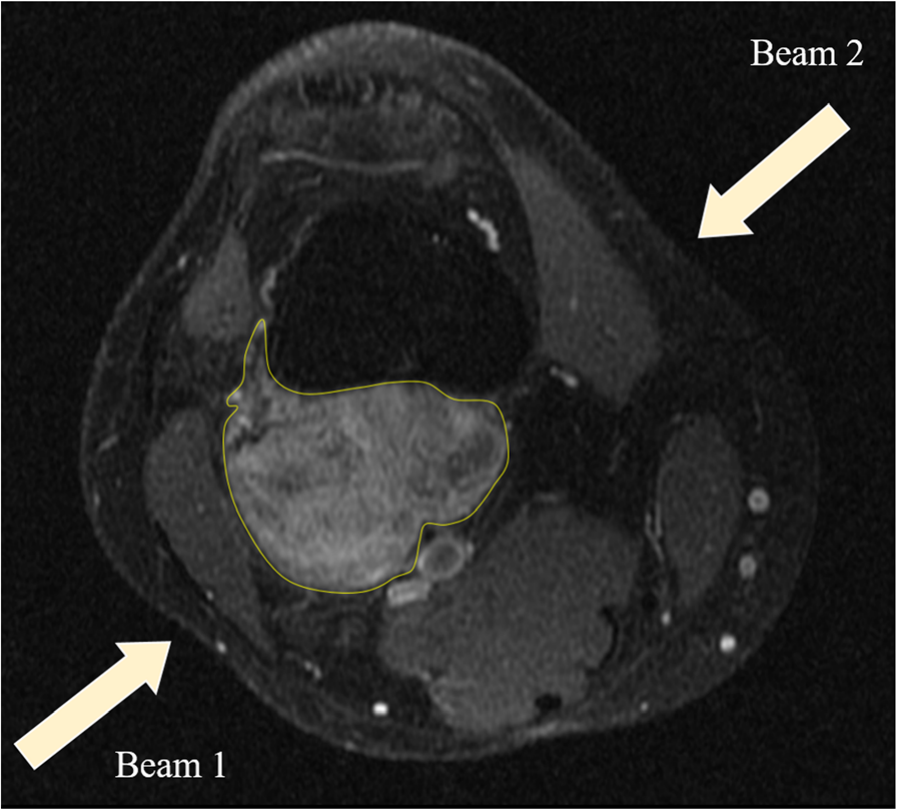
MRI image of the osteosarcoma tested with the selected beam configuration

The medical images have been used as an input for NCTPlan [29], to create a voxelized model of the limb in MCNP syntax. The most efficient irradiation configuration consisted in two parallel opposing fields (Fig. 2). The dosimetry of the tumour and the surrounding tissues has been then assessed with the 12 cm diameter accelerator beam. The beam design used in this work is the result of the first optimization of the BSA to produce a suitable dose distribution in tumour and in normal surrounding tissues.

The prescription was to deliver a maximum dose to the skin to 22 Gy-Eq, calculated with the traditional formalism of fixed RBE and CBE factors obtained by UMR-106 cell survival curves.

In the photon equivalent dose formalism, the biological weighting factors are taken at fixed end-points, such as 1% of survival in in-vitro experiments. Iso-effective dose model exploits the entire curve of cell survival as a function of the dose instead of fixing an arbitrary end-point to calculate RBE/CBE. The survival curves are used to find the dose that equals the Tumour Control Probability (TCP) obtained with BNCT and with photon therapy. In this way, it is possible to generate values of iso-effective dose, that is *the photon dose that produces the same cell survival level as the combination of the different BNCT dose components*. The parameters to calculate photon iso-effective dose were calculated from the UMR-106 cell survival curves and used in the Sphere software developed in CNEA [30].

To understand whether the dosimetry obtained was potentially therapeutic, the simulated treatment was compared with a case of osteosarcoma treated by BNCT at KURRI in Japan [20]. The patient was a 54-year-old Japanese female with recurrent radiation-induced osteosarcoma of the skull (tumour volume 169 cm^3^). The tumour was very aggressive, with subcutaneous and epidural extension and it was inoperable and radioresistant. After BNCT the subcutaneous-epidural tumour reduced with no adverse effect and the general conditions of the patient improved very rapidly. The dosimetry of this case was taken as a reference to evaluate the performance of the RFQ beam on the analysed clinical case. The minimum-maximum and median tumour dose values were re-calculated both using RBE/CBE factors obtained in Pavia and also with iso-effective dose formalism.

## Results

### Osteosarcoma cell survival

**Fig. 3:**
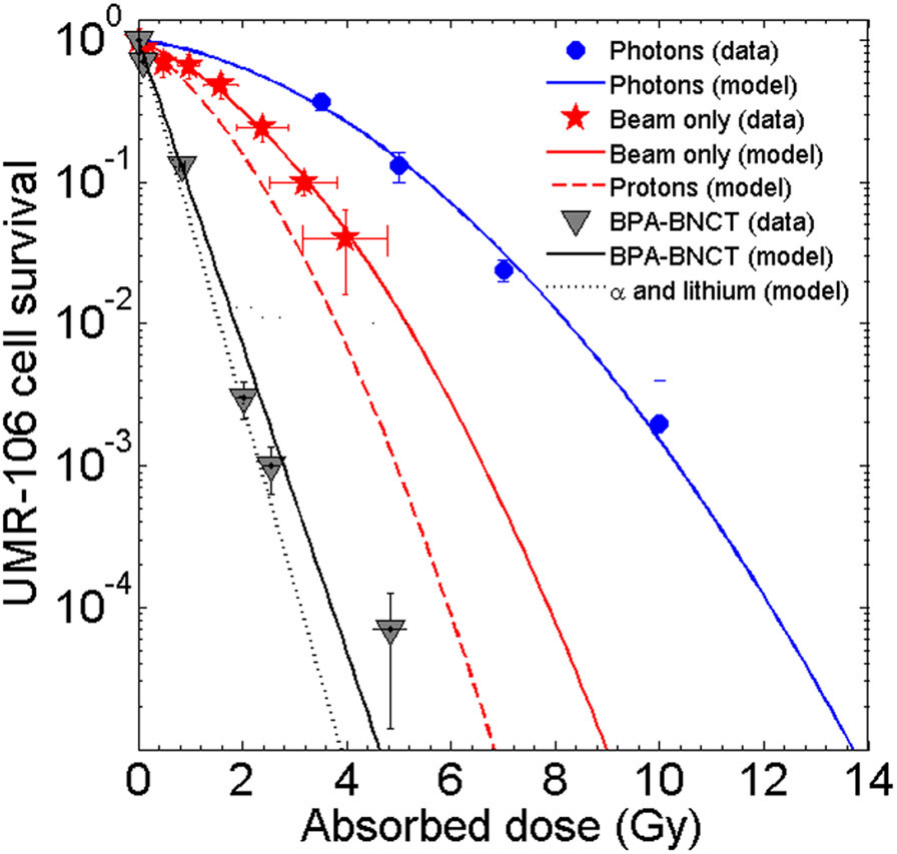
Rat osteosarcoma UMR-106 cell survival curves as a function of the absorbed dose. Experimental values correspond to data published in [21], and the new measurements carried out in this work

Figure 3 depicts the UMR-106 cell survival data along with the fit for the photon reference radiation, the neutron beam-only component (without presence of boron) and the neutron beam with boron. Reported experimental values account for data published in previous studies [21], and for new experiments carried out in this work. The uncertainty of the dose values in the beam-only curve is dominated by the error of the boron concentration measured, in the order of 1 ppm, close to the sensitivity limit of the measurement technique.

The fit was obtained by models described in [18], that take into account first order lesion repair by means of the Lea-Catcheside time factor, assuming that the repair kinetics follows a bi-exponential decline with fast and slow characteristic repair times [31, 32] and synergism between different radiation components. See Appendix for the details. The fit for the reference radiation provides α_Ref_ and β_Ref_ listed in Table 1. Note that α_Ref_/β_Ref_ is about two, that is low compared with usual values used for tumour, but in agreement with α/β ratios reported in literature for sarcomas [33]. In addition, the fit for the beam-only and for the BNCT was calculated simultaneously for the two curves as explained in [18], resulting in a set of parameters (α_n_, β_n_, α_B_) listed in Table 1, together with the 95% confidence interval (95% CI). As a first attempt, a 4 parameter model was used to fit the curves. The results showed that the parameter β_B_ for the boron component was virtually zero, indicating that the quadratic term of the boron component can be neglected, thus reducing the number of free parameters to 3. The fit of the gamma component led to the parameters α_Ref_, β_Ref_ for the reference radiation component. These parameters were used for the iso-effective dose calculation.

**Table 1:** Radiobiological parameters of the cell survival curves of Fig. 3 (modified linear quadratic model). Ref denotes photon component, n neutrons component and B boron component

Parameter	Value [95% CI]
***α***_***Ref***_ [Gy^**−**1^]	0.120 [−0.003 – 0.243]
***β***_***Ref***_ [Gy^**−**2^]	0.05 [0.03 – 0.08]
***α***_***n***_ [Gy^**−**1^]	0.62 [0.28 – 0.95]
***β***_***n***_ [Gy^**−**2^]	0.16 [0.04 – 0.37]
***α***_***B***_ [Gy^**−**1^]	3.0 [2.4 – 3.6]

Dose-survival curves that result considering protons only (beam-only without gamma component) and boron reaction only (BPA-BNCT without the contribution of protons and gamma) are also depicted in Fig. 3. These curves are used to compute RBE and CBE factors for 1% cell survival. Results are listed in Table 2, together with the assumed values for the skin, taken from in vivo radiobiological experiments [28, 34].

**Table 2:** One percent survival RBE and CBE values used for equivalent dose calculation. Skin values are taken from literature [28, 34], osteosarcoma ones from UMR-106 in vitro experiments

	RBE (protons)	RBE (photons)	CBE
Skin	2.5	1	2.5
Osteosarcoma	2.2 ± 0.5^a^	1	5.3 ± 1.5^a^

^a^ Error taking into account 95% CI of the dose values at 0.01 survival level for the three components

### Boron concentrations in tissues

**Fig. 4:**
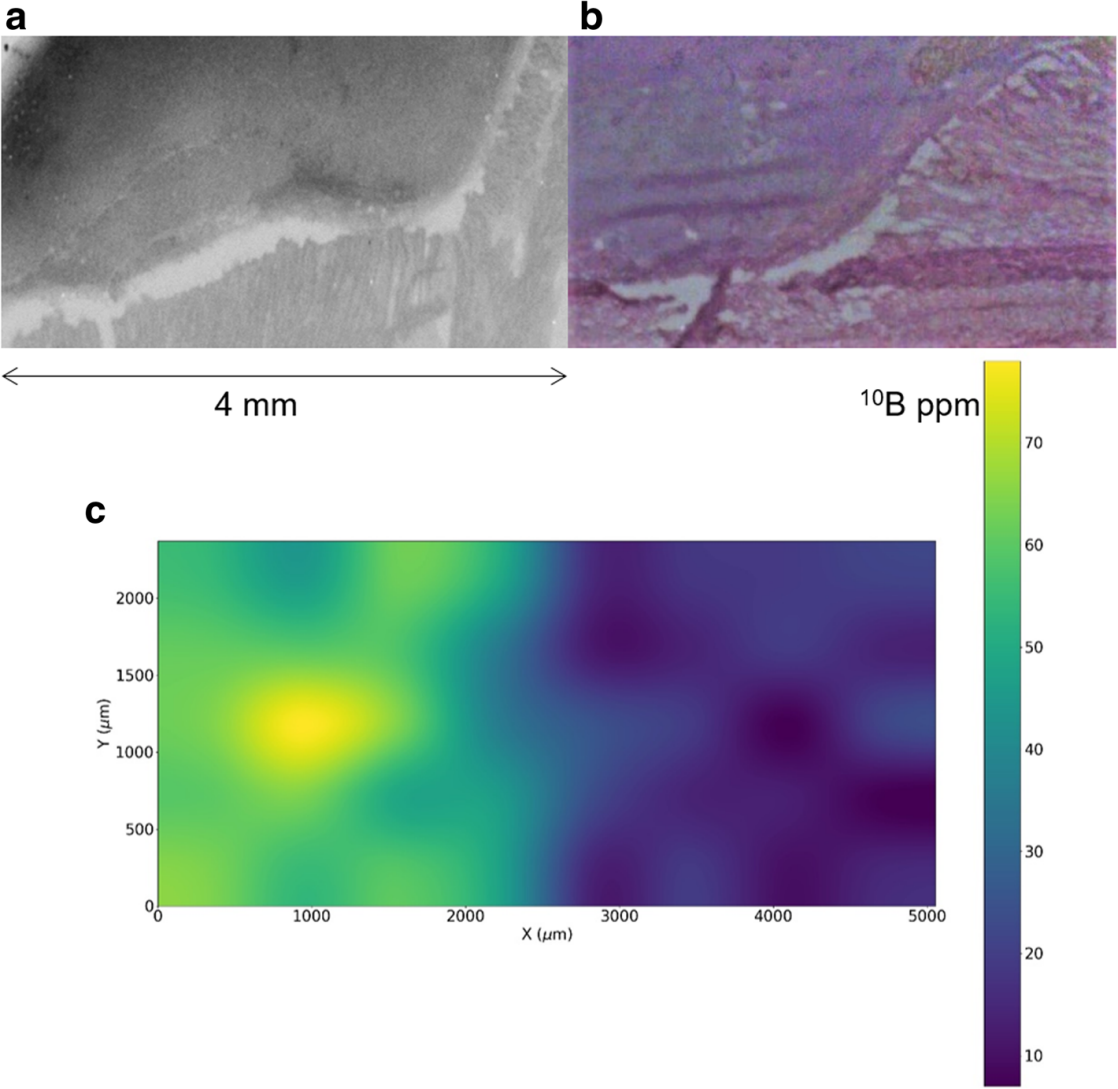
**a** qualitative neutron autoradiography of a section taken from a tumour biopsy (rat 1). **b** histological preparation of a subsequent section showing that the tumour is confined in the left part. **c** map of boron distribution obtained by quantitative neutron autoradiography in a third section. Higher boron concentration is clearly in the left part, where the tumour is. However, boron concentration is not uniform, due to the fact that tumour tissue is non homogeneous

Measurements and imaging by neutron autoradiography showed that ^10^B is more concentrated in the tumour than in the healthy surrounding tissues, especially in those cases where the animal model developed tumour volumes with clear presence of viable cells. The analysis of the healthy muscle was more straightforward because boron distribution was more uniform, as expected. The average boron concentration in healthy muscle measured in 7 rats is 15.4 ppm with a standard deviation of 4.0 ppm, thus 15 ppm was taken as the reference for normal tissues in the dosimetry calculations. In tumour, the analysis was more complex, due to the non-homogeneous nature of tissue and the consequent variability of boron concentration values. As an example, the selectivity of BPA uptake in the tumour area is shown in Fig. 4 where a neutron autoradiography image (a) is compared with a histological preparation of a contiguous tissue section (b). Darker areas correspond to the most vital part of the tumour. Figure 4c is the quantitative map of boron distribution in part of the sample, where colours represent concentration in ppm. The high non-uniformity of boron distribution in tumour is evident. However, the most viable parts of tumour uptake boron up to concentration of about 75 ppm. This kind of analysis was performed in three rats showing vital tumour, and selecting the areas where the osteosarcoma was not necrotic nor characterized by haemorrhage, the average value found was 64 +/−18 ppm. These results, although not constituting a complete boron bio-distribution study in osteosarcoma and despite the unevenness of concentration measured in the tumour area due to the heterogeneity of tissues, nevertheless show that it is possible to obtain a boron concentration ratio comparable to 3.5, obtained in clinical BNCT and in other animal studies [19, 20]. Basing on these results, we chose a representative value in tumour of 60 ppm for the treatment planning simulations.

Table 3 reports the boron concentrations used for dose calculations in the present work.

**Table 3:** Boron concentrations used in dose calculation

Tissue type	
Osteosarcoma	60 ppm
Healthy muscle/bone	15 ppm
Skin	22.5 ppm

Some published experimental data on BPA uptake in other sarcomas may be compared with our results. In particular, works on human clear cell sarcoma (CSS) implanted in nude mice demonstrated that there is a selective uptake of boron and that irradiation causes the selective destroy of the tumour with no side effects in the other tissues [35, 36]. Authors measured a tumour-to-skin concentration ratio of about 4.5 at 1 h after BPA administration. This value is higher than what normally assumed for tumours that are currently treated in clinical trials such as glioblastoma, head and neck cancers and melanoma, for which the ratio commonly employed for dose calculation is 3.5, and compatible to our findings (tumour-to-muscle concentration ratio equal to 4, tumour-to-skin concentration ratio assumed equal to 2.7).

### RBE-weighted dose VS photon iso-effective dose

**Fig. 5:**
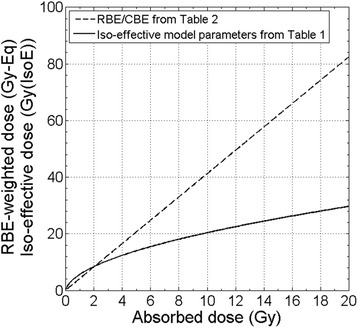
RBE/CBE-weighted dose (*dashed line*) compared to iso-effective dose (*solid line*) as a function of absorbed dose, using RBE factors and model parameters derived from experimental UMR-106 survival curves

Figure 5 shows the difference between RBE-weighted dose and photon iso-effective dose as a function of absorbed dose, using the RBE/CBE factors, the survival parameters and boron concentrations obtained from the in-vitro and in-vivo experiments. The proportion of the components of the total absorbed dose used for the calculations are the ones of the accelerator beam at the tumour position. For low absorbed doses (i.e., lower than 3 Gy) the RBE-weighted and the iso-effective doses are almost equal. However, for absorbed doses typically delivered to tumour, the difference is significant: at 10 Gy, the RBE-weighted dose is nearly 40 Gy-Eq, while iso-effective dose is around 20 Gy (IsoE).

### Treatment planning simulations

**Fig. 6:**
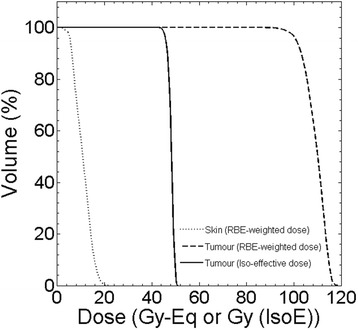
Dose Volume Histograms for skin and tumour calculated with RBE and iso-effective formalisms

The results of the treatment planning simulations with the RFQ beam based on the RBE and iso-effective formalism are shown in Fig. 6. The prescription to deliver a maximum dose to the skin equal to 22 Gy-Eq led to a treatment time of 47 min (23.5 min per beam).

The picture shows the cumulative Dose Volume Histograms (DVH) for skin and tumour. For skin, only one DVH is shown because the iso-effective parameters for healthy tissues are not available yet. Then, the same treatment time was used to calculate the two DVH for tumour. If the iso-effective model together with the UMR-106 survival parameters was assumed valid for skin, the iso-effective dose would be 20% lower than the prescription RBE-weighted dose. Thus, the choice of 47 min as a treatment time also for iso-effective dose calculation is conservative in this case. The two DVH for tumour show a good level of uniformity for a BNCT treatment, proving that two parallel opposing neutron beams from the RFQ accelerator would ensure a good tumour dose coverage.

Table 4 reports the minimum, maximum and mean tumour dose for the two formalisms. As expected, the iso-effective doses are almost half the RBE-weighted values.

**Fig. 7:**
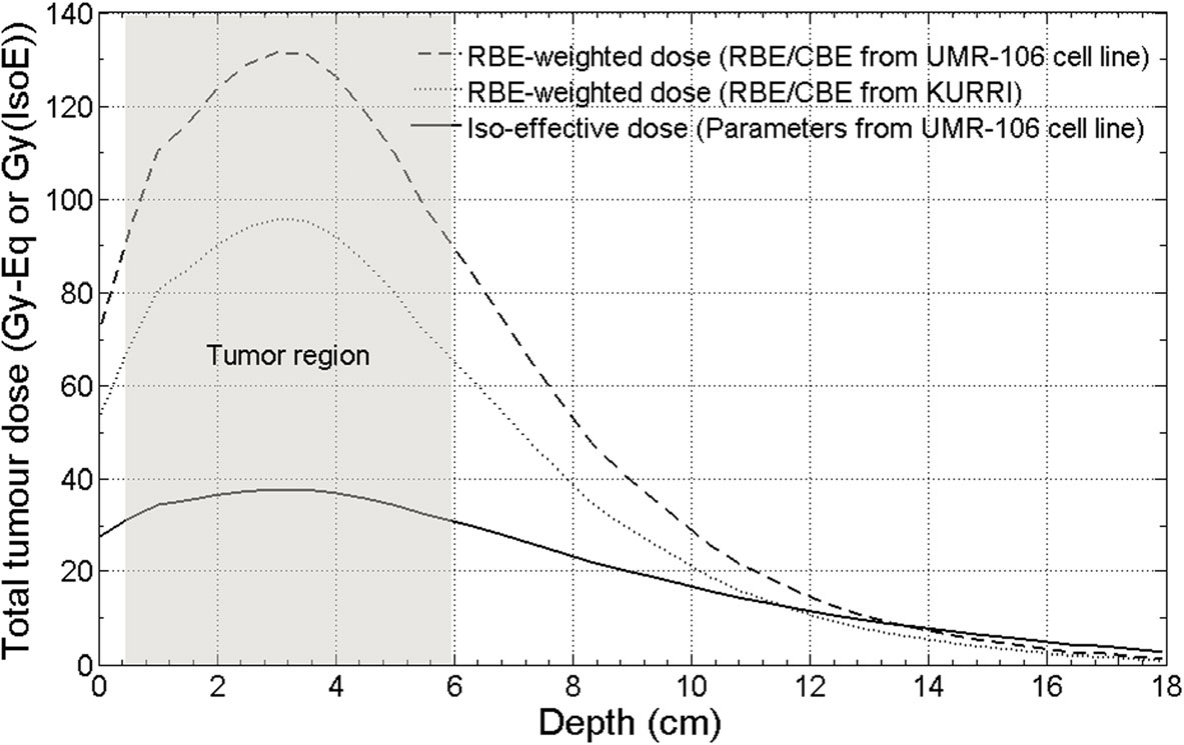
Profile of the tumour dose in the patient along the neutron beam central axis: RBE-weighted dose with UMR-106 factors (Table 2), RBE-weighted dose with KURRI factors, and photon iso-effective dose with UMR-106 cell survival parameters (Table 1)

Figure 7 shows the tumour dose profile in Japanese patient along the central axis of the neutron field. The three plotted dose curves are calculated with the RBE/CBE factors reported in Table 2, the original RBE/CBE factors used in KURRI (i.e., 3.0 for neutrons, 3.8 for boron, 1.0 for photons), and the iso-effective parameters listed in Table 1.

## Discussion

As other types of radiotherapy, BNCT needs to take into account the radio-sensitivity of the tissues and organs involved in the irradiation. Fast neutron radiotherapy has been used to treat different types of sarcomas, such as soft tissue sarcomas and osteosarcomas [37–40]. Although the local control rate with this therapy was encouraging, the incidence of late complications was sometimes considered unacceptably high as reported in the cited papers. A trial for soft-tissue sarcoma was stopped because of severe complications in the treated patients, including early damage (erythema, mucositis, dry or moist desquamation developing within 3 months of commencement of irradiation) and late damage (fibrosis, telangiectasia or tissue necrosis) [41]. Reported complications for sarcomas involved predominantly the skin and were related to the high neutron doses delivered and/or the large radiotherapy fields used. The presence of hot spots in dose distribution to the skin has been identified as a source of severe side effects. Compared to fast neutron therapy, BNCT has the potential to reduce the dose to the skin and to the other healthy tissues because, as mentioned before, its selectivity strongly depends on boron bio-distribution rather than on the neutron beam.

Indeed, in clinical BNCT dose is prescribed to the most sensitive organ at risk, considering that tumour receives a higher dose due to the differential boron uptake. From literature, certain levels of toxicity have been reported, along with the protocol to handle the adverse effects and the final results of the treatment. BNCT has been applied especially to brain and head and neck cancer, however, to make predictions about the possible toxicity that may derive from osteosarcoma BNCT, the relevant examples are BNCT of skin melanoma in Argentina, where limbs were irradiated at the RA-6 reactor with hyper-thermal neutrons [42] and, of course, BNCT of skull osteosarcoma. In the first case, the organ at risk is the skin, thus it is a good reference also for limb osteosarcoma BNCT. The cited paper reports an acceptable toxicity, with 7 out of 10 evaluable areas showing a very mild erythema (G1) and 3 out of 10 moist desquamation and ulceration that in all the cases was solved with medical care and medication (30% toxicity grade 3). In particular, the most severe adverse effects were observed in the skin of the foot, an anatomic area that in case of osteosarcoma would be spared from irradiation. Also in the case of BNCT for skull osteosarcoma no radiation injury of the scalp was caused, the only adverse effect being hair loss in neutron field [20]. This experience also gives information about possible effects in normal bone: in this case dose was limited to the normal brain, but thanks to the tumour-to-normal bone boron concentration ratio, the skull was not damaged. The other published experience of BNCT for tumours infiltrated in normal bone [19] show that a severe side effect can be indeed produced to the normal bone: Case 5 reported in the cited paper had radio-osteonecrosis that required surgery. In the same paper, however, Case 2 who was treated for a maxillary osteosarcoma, showed no adverse effect in normal bone. These findings push further studies on boron distribution in normal bone in order to evaluate the dose in a more refined way for each patient. In this sense, more bio-distribution studies in animals and PET with F18-BPA will be the response in a single-patient based study.

A natural question arising is how the BNCT results obtained in this work can be compared with reported clinical radiation therapy in osteosarcoma. In literature, a number of protocols of osteosarcoma irradiation with photons is reported, ranging from conventional fractionated schemes to extremely high single photon doses delivered by intraoperative radiotherapy or extracorporeal irradiation. Shwartz et al., analysed the outcomes of irradiated patients in the Cooperative Osteosarcoma Study Group (COSS)-Registry, including 100 osteosarcoma patients that received radiotherapy for curative or for palliation intent [43]. The dose distribution of the different fractionated protocols included in the analysis, were described as: dose for external RT was 55.8 Gy (30–120); for preoperative RT, 50 Gy (30–68); postoperative RT, 54 Gy (36–72); for RT without surgery, 56 Gy (30–75,6); for primary RT, 59.7 Gy (20–120); for local recurrence, 50.4 Gy (30–70); and for metastases, 45 Gy (30–66). The conclusions of this review were that a clear dose-response relationship could not be demonstrated. However, in the same work it is mentioned that Machak et al. [44] and in a number of patients treated by Shwartz, long-term local control after fractionated doses of 60 Gy and chemotherapy could be demonstrated. Moreover, Gaitan-Yanguas et al. [45] showed that there was no control below 30 Gy, and 100% control above 90 Gy. Based on these experiences, a possible starting point to compare BNCT single fraction dose with the mentioned protocols, is that a fractionated dose of at least 90 Gy would be necessary to achieve complete local control. A dose of 90 Gy in a 2 Gy/fraction scheme corresponds to a single dose of about 15 Gy using Linear Quadratic Model and the α_Ref_, β_Ref_ obtained for UMR-106 cell survival curve (Table 1). Then, regardless of the formalism employed to calculate doses both in limb (Fig. 6) and in skull osteosarcoma (Fig. 7), the dose distributions obtained by BNCT would be potentially therapeutic to treat this disease.

Regarding the single fraction schemes, the reported delivered photon doses are quite variable. Aporchayanon et al. irradiated 21 osteosarcoma patients with bone auto-transplantation delivering 100 Gy [9]; Mottard et al. delivered 70 or 90 Gy with the same technique [46]. Both authors obtained excellent local control. In addition, intraoperative irradiation was carried out in some centres, using for example 10-20 Gy of photons in children and adolescents [47], or 45-80 Gy of photons including also adult patients [7]. Again, BNCT dose values calculated with both formalisms fall in this wide range of single photon doses, RBE-weighted doses being even higher.

In papers describing clinical radiotherapy of osteosarcoma, the main effects limiting the dose to be administrated to the limb are the pathologic bone fracture risk and the tolerance of major nerves and vessels. Unlike for photon therapy, BNCT dose distribution is selective for tumour, thus normal bone absorbs lower dose. In particular, for the analysed femur osteosarcoma, the mean absorbed dose in normal bone (100 cm^3^) is 15.4 Gy-Eq (minimum 11 Gy-Eq, maximum 18.7 Gy-Eq), a value considerably lower of the single dose administered in intraoperative or extracorporeal bone irradiation with photons.

Oya et al. report that in most intraoperative photon therapy for osteosarcomas in the extremities, the delivered dose was limited to 20 Gy in a single fraction to the tumour bed in order to spare major vessels, nerves, and surrounding soft tissue [7]. Their dose escalation was possible because they excluded nerves and vessels from the irradiation field. Dose distribution obtained with BNCT in the neurovascular bundle falls between a minimum dose of 14 Gy-Eq and a maximum of 18 Gy-Eq, with a mean value of 16 Gy-Eq, calculated with an RBE of 2.5 and a CBE equal to 1.3, as assumed for the central nervous system in the treatment of the brain tumours. These values are safe according to the published tolerance dose of femoral arteries, that is as high as 30 Gy in single fraction [48], and of peripheral nerve, that is 20 Gy for intraoperative radiotherapy [49].

With the aim to understand if calculated doses in the femur case are potentially therapeutic, the dosimetry of the femur case was analysed in the light of the Japanese clinical case re-calculated in this work with the new osteosarcoma parameters and the iso-effective formalism. It can be seen that the maximum dose obtained in limb osteosarcoma using the accelerator (Table 4) is as high as the maximum RBE-weighted doses calculated with the two sets of RBE/CBE factors in skull osteosarcoma. The same holds for the minimum dose in limb osteosarcoma (87 Gy-Eq) when compared to the minimum of KURRI (67.7 Gy-Eq) and to the one calculated with RBE/CBE from UMR-106 cell line (92 Gy-Eq). Note that while the minimum dose in limb is the absolute minimum value in the whole tumour volume, the minimum value in skull corresponds to the lowest dose in the central beam profile (at 5.8 cm from the patient surface). Therefore, given that the patient showed a very good local tumour control, these results support that the same outcome could be obtained in limb with the accelerator beam. The iso-effective dose profile shows values sensibly lower than RBE-weighted dose: maximum iso-effective dose in patient is about 37.8 Gy (IsoE), minimum in tumour along the profile is 31.5 Gy (IsoE). It is worth mentioning the tumour was reduced dramatically without radiation injury of the scalp, therefore the same outcome can be expected in the femur osteosarcoma case.

**Table 4:** Results of dose distribution in femur osteosarcoma with the two formalisms. Dose prescription: 22 Gy-Eq in skin, irradiation time: 47 min

RBE-weighted dose (Gy-Eq) Mean [Min-Max]	Iso-effective dose (Gy (IsoE)) Mean [Min-Max]
109.2 [87.0 -118.4]	48.2 [43.2 - 51.0]

## Conclusions

This work explores the feasibility of BNCT for osteosarcoma using a neutron beam from a proton accelerator, from the point of view of the dose distribution. A human parosteal osteosarcoma of the distal femur was used as an example to calculate treatment planning. Dose was calculated both using traditional formalism of RBE-weighted dose and the new formalism of photon iso-effective dose that proved to give robust results in BNCT of melanoma and cerebral tumours. For the first time, survival curves for photons, neutrons and BNCT were obtained for an osteosarcoma cell line, allowing the derivation of the RBE/CBE factors and of the parameters for the iso-effective dose in osteosarcoma. Moreover, boron concentration values were measured in vivo in a small animal model bearing this tumour.

The results show that the dose distribution obtained in femur osteosarcoma is potentially therapeutic if compared to the photon therapy protocols reported in literature and at least as good as the BNCT dose delivered to the radio-induced skull osteosarcoma in Japan. This supports the suitability of an accelerator-based neutron beam peaked between 1 and 10 keV for the treatment of deep-seated bone tumours.

Several authors explain that osteosarcoma is a radio-resistant tumour not because of the sensitivity of tumour cells to radiation, but rather due to factors such as hypoxia, reoxygenation, tumor size, repair and proliferation rate, that limit the effectiveness of photon therapy [36, 47]. In particular, tumour cell repair of sublethal radiation damages may play a role in the poor outcome of standard radiotherapy [44]. These elements are much less crucial in the case of BNCT that is characterized by high-LET radiation with localized energy deposition inside tumour cells, and that may thus represent a more effective option to treat this disease.

The possibility to apply BNCT to osteosarcoma would allow a multimodal treatment consisting in neo-adjuvant chemotherapy, high-LET selective radiation treatment and a more conservative surgery followed by adjuvant chemotherapy.

## Appendix

Let *α*_*R*_ and *β*_*R*_ be the coefficients of the single fraction survival model for the photon reference radiation *S*_*R*_, and let *G*_*R*_(*θ*) denote the generalized Lea-Catcheside time factor for the irradiation time *θ* [31]. Then, the assumed expression for *S*_*R*_ is the generalized linear-quadratic model that accounts for dose rate dependent sublesion repair

$$ {S}_R\left({D}_R\right)={e}^{\left(-\left({\alpha}_R{D}_R+{G}_R\left(\theta \right){\beta}_R{D_R}^2\right)\right)}, $$

with *D*_*R*_ the dose of the photon reference radiation *R*.

Let the repair kinetics follow a bi-exponential decline with fast and slow characteristic repair times *t*_0*f*_ and *t*_0*s*_ independent of LET [32]. Schmid et al. [32] noticed that, while the fast and slow component have identical half-life values for different radiation qualities, apparent differences in the kinetics following high and low LET irradiation could completely be attributed to quantitative differences in their contributions, with the slow component being responsible for 47% of the repair after exposure to low LET radiation as compared to 80% after high LET radiation. Based on this approach, the expression for the Lea-Catcheside time factor is given by:

$$ {G}_R\left(\theta,,, {t_0}_f,{t_0}_s\right)={a_R}_fG\left(\theta, {t_0}_f\right)+{a_R}_sG\left(\theta, {t_0}_s\right), $$

where *a*_*Rf*_ and *a*_*Rs*_ are the proportions of sublesions repaired by the fast and slow kinetics for radiation R (with *a*_*Rf*_ + *a*_*Rs*_ = 1), and

$$ G\left(\theta, {t}_0\right)=\frac{2{t}_0}{\theta}\left(1-\frac{t_0}{\theta}\left(1-{e}^{-\frac{\theta }{t_0}}\right)\right) $$

is the time factor for simultaneous build up and repair of radiation damage, considering an irradiation at a constant dose rate. Note that for these irradiation conditions, the logarithm of the survival given by the generalized LQ model behaves linearly at high doses, approaching a constant slope as the dose increases. Therefore, in opposition to the simple LQ model, the assumed model adequately represents the high dose region, a fact of special importance in BNCT since the doses that are to be translated into single fraction photon doses are typically beyond the survival experimental data.

Following the results reported in [32], the fast and slow characteristic repair times *t*_0*f*_ and *t*_0*s*_ and the proportions of the sublesions repaired by the two kinetics *a*_*Rf*_ and *a*_*Rs*_ for the reference low LET radiation were taken as 24/ln2 min and 14/ln2 h, and 0.53 and 0.47, respectively,

Let *D*_1_ ,  …  , *D*_4_ be the four absorbed dose components of the mixed field BNCT radiation. The assumed survival model for this case *S*(*D*_1_,  … , *D*_4_) takes into account synergistic interactions between the different radiations and it is expressed, as a function of the total absorbed dose ${D}_T={\sum}_{i=1}^4{D}_i$ and the relative contribution of each dose component *f*_*i*_ = *D*_*i*_/*D*_*T*_, as

$$ -\ln \left(S\left({D}_1,\dots, {D}_4\right)\right)=\left(\sum_{i=1}^4{\alpha}_i{f}_i+\sum_{i=1}^4\sum_{j=1}^4{G}_{ij}\left(\theta \right)\sqrt{\beta_i{\beta}_j}{f}_i{f}_j\right){D_T}^2, $$

where *α*_*i*_ and *β*_*i*_ are the coefficients of the single fraction generalized LQ model, and *G*_*ij*_(*θ*) the time factor for a simultaneous mixed irradiation that accounts for the repair of pairs of sublesions produced by radiations *i* and *j* during the irradiation time *θ*.

If production of one of the sublesions does not affect the production of the other one, it can be shown that *G*_*ij*_(*θ*) can be expressed as a sum of independent *G* factors, i.e., *G*_*ij*_(*θ*) = *a*_*i*_ *G*_*i*_(*θ*) + *a*_*j*_ *G*_*j*_(*θ*), each one weighted by the relative proportion between components *a*_*i*_ and *a*_*j*_, with *a*_*i*_ + *a*_*j*_ = 1 [18]. To compute expressions *G*_*ij*_, the proportions of the sublesions repaired by the two kinetic *a*_*if*_s and *a*_*is*_ were taken as 0.57 and 0.43 for the low LET radiation (i.e., for *i* = 4), and 0.2 and 0.8, for the high LET radiations (i.e., for *i* = 1,2,3) [32].

## Abbreviations

^10^B: 10-boron
BNCT: Boron neutron capture therapy
BPA: Boronophenylalanine
BSA: Beam shaping assembly
CNEA: Comisión Nacional de Energía Atómica
DVH: Dose volume histogram
INFN: Istituto Nazionale di Fisica Nucleare
KURRI: Kyoto University Research Reactor Institute
LET: Linear energy transfer
MRI: Magnetic resonance imaging
RBE/CBE: Relative Biological Effectiveness/Compound Biological Effectiveness
RFQ: Radio frequency quadrupole
TCP: Tumour control probability

## References

[1] Bielack S, Carrle D, Casali PG, ESMO guidelines working group (2009). Osteosarcoma: ESMO clinical recommendations for diagnosis, treatment and follow-up. Ann Oncol.

[2] Luetke A, Meyers PA, Lewis I, Juergens H (2014). Osteosarcoma treatment – where do we stand? A state of the art review. Cancer Treat Rev.

[3] Geller DS, Gorlick R (2010). Osteosarcoma: a review of diagnosis, management, and treatment strategies. Clin Adv Hematol Oncol.

[4] Bacci G, Ferrari S, Mercuri M, Bertoni F, Picci P, Manfrini M, Gasbarrini A, Forni C, Cesari M and Campanacci M, Predictive factors for local recurrence in osteosarcoma 540 patients with extremity tumors followed for minimum 2.5 years after neoadjuvant chemotherapy. Acta Orthopaedica Scandinavica, 1998;69(3):230–236 10.3109/17453679809000921 9703394

[5] Grimer RJ, Taminiau AM, Cannon SR (2002). Surgical outcomes in osteosarcoma. J Bone Joint Surg (Br).

[6] Yasko AW (2009). Surgical management of primary osteosarcoma. Cancer Treat Res.

[7] Oya N, Kokubo M, Mizowaki T, Shibamoto Y, Nagata Y, Sasai K, Nishimura Y, Tsuboyama T, Toguchida J, Nakamura T, Hiraoka M (2001). Definitive intraoperative very high-dose radiotherapy for localized osteosarcoma in the extremities. Int J Radiat Oncol Biol Phys.

[8] Blattmann C, Oertel S, Schulz-Ertner D, Rieken S, Haufe S, Ewerbeck V, Unterberg A, Karapanagiotou-Schenkel I, Combs SE, Nikoghosyan A, Bischof M, Jäkel O, Huber P, Kulozik AE, Debus J (2010). Non-randomized therapy trial to determine the safety and efficacy of heavy ion radiotherapy in patients with non-resectable osteosarcoma. BMC Cancer.

[9] Arpornchayanon O, Leerapun T, Pruksakorn D, Panichkul P (2013). Result of extracorporeal irradiation and re-implantation for malignant bone tumors: a review of 30 patients. Asia Pac J Clin Oncol.

[10] Barth RF, Vicente MG, Harling OK, Kiger WS, Riley KJ, Binns PJ (2012). Current status of boron neutron capture therapy of high grade gliomas and recurrent head and neck cancer. Radiat Oncol.

[11] Yoshino K, Suzuki A, Mori Y, Kakihana K, Honda C, Mishima Y, Kobayashi T, Kanda K (1989). Improvement of solubility of pboronophenylalanine by complex formation with monosaccharides, Strahlenther. Onkol Organ Dtsch Rontg.

[12] González SJ, Bonomi MR, Santa Cruz GA, Blaumann HR, Larrieu OAC, Menéndez P (2004). First BNCT treatment of a skin melanoma in Argentina: dosimetric analysis and clinical outcome. Appl Radiat Isot.

[13] Suzuki M, Sakurai Y, Masunaga S, Kinashi Y, Nagata K, Maruhashi A (2006). Feasibility of boron neutron capture therapy (BNCT) for malignant pleuralmesothelioma from a viewpoint of dose distribution analysis. Int J Radiat Oncol.

[14] Mitsumoto T, Yajima S, Tsutsui H, Ogasawara T, Fujita K, Tanaka H, Sakurai Y, Maruhashi A (2013). Cyclotron-based neutron source for BNCT. AIP Conference Proceedings.

[15] Esposito J, Colautti P, Fabritsiev S, Gervash A, Giniyatulin R, Lomasov VN, Makhankov A, Mazul I, Pisent A, Pokrovsky A, Rumyantsev M, Tanchuk V, Tecchio L (2009). Be target development for the accelerator-based SPES-BNCT facility at INFN Legnaro. Appl Radiat Isot.

[16] Farías RO, Bortolussi S, Menéndez P, González SJ (2014). Exploring boron neutron capture therapy for non-small cell lung cancer. Physica Medica.

[17] Coderre JA, Makar MS, Micca PL, Nawrocky MM, Liu NB, Joel DD, Slatkin DN, Amols HI (1993). Derivations of relative biological effectiveness for the high-let radiations produced during boron neutron capture irradiations of the 9l rat gliosarcoma *in vitro* and *in vivo*. Int J Radiat Oncol Biol Phys.

[18] González SJ, Santa Cruz GA (2012). The photon-isoeffective dose in boron neutron capture therapy. Radiat Res.

[19] Kato I, Ono K, Sakurai Y, Ohmae M, Maruhashi A, Imahori Y, Kirihata M, Nakazawa M, Yura Y (2004). Effectiveness of BNCT for recurrent head and neck malignancies. Appl Radiat Isot.

[20] Futamura G, Kawabata S, Siba H, Kuroiwa T, Suzuki M, Kondo N, Ono K, Sakurai Y, Tanaka M, Todo Tand Miyatake S (2014). A case of radiation-induced osteosarcoma treated effectively by boron neutron capture therapy. Radiat Oncol.

[21] Cansolino L, Clerici AM, Zonta C, Dionigi P, Mazzini G, Di Liberto R, Altieri S, Ballarini F, Bortolussi S, Carante MP, Ferrari M, González SJ, Postuma I, Protti N, Santa Cruz GA, Ferrari C (2015). Comparative study of the radiobiological effects induced on adherent vs suspended cells by BNCT, neutrons and gamma rays treatments. Appl Radiat Isot.

[22] Postuma I, Bortolussi S, Protti N, Ballarini F, Bruschi P, Ciani L, Ristori S, Panza L, Ferrari C, Cansolino L, Altieri S (2016). An improved neutron autoradiography set-up for 10B concentration measurements in biological samples. Rep Pract Oncol Radiother.

[23] Goorley T, James M, Booth T, Brown F, Bull J, Cox LJ, Durkee J, Elson J, Fensin M, Forster MA, Hendricks J, Hughes HJ, Johns R, Kiedrowski B, Martz R, Mashnik S, McKinney G, Pelowitz D, Prael R, Sweezy J, Waters L, Wilcox T, Zukaitis T (2012). Initial MCNP6 release overview. Nucl Technol.

[24] Ferrari C, Zonta C, Cansolino L, Clerici AM, Gaspari A, Altieri S, Bortolussi S, Stella S, Bruschi P, Dionigi P, Zonta A (2009). Selective uptake of p-boronophenylalanine by osteosarcoma cells for boron neutron capture therapy. Appl Radiat Isot.

[25] Bortolussi S, Altieri S (2013). Boron concentration measurement in biological tissues by charged particles spectrometry. Radiat Environ Biophys.

[26] Altieri S, Bortolussi S, Bruschi P, Chiari P, Fossati F, Stella S, Prati U, Roveda L, Zonta A, Zonta C, Ferrari C, Clerici A, Nano A, Pinelli T (2008). Neutron autoradiography imaging of selectiveboron uptake in human metastatic tumours. Appl Radiat Isot.

[27] Farías RO, Garabalino MA, Ferraris S, Santa María J, Rovati O, Lange F, Trivillin VA, Monti Hughes A, Pozzi ECC, Thorp SI, Curotto P, Miller ME, Santa Cruz GA, Bortolussi S, Altieri S, Portu AM, Saint Martin G, Schwint AE, González SJ (2015). Towards a clinical application of ex-situ boron neutron capture therapy for lung tumors at the RA-3 reactor in Argentina. Med Phys.

[28] Fukuda H, Hiratsuka J, Honda C, Kobayashi T, Yoshino K, Karashima H, Takahashi J, Abe Y, Kanda K, Ichihashi M (1994). Boron neutron capture therapy of malignant melanoma using 10b-paraboronophenylalanine with special reference to evaluation of radiation dose and damage to the normal skin. Radiat Res.

[29] González SJ, Carando DG, Santa Cruz GA, Zamenhof RG (2005). Voxel model in BNCT treatment planning: performance analysis and improvements. Phys Med Biol.

[30] Gossio S, Carando DG, González SJ (2009). A computational Dosimetry tool for the study of tumor doses and skin toxicities in BNCT. Appl Radiat Isot.

[31] Lea DE, Catcheside DG (1942). The mechanism of induction by radiation of chromosome aberrations in Tradescantia. J Genet.

[32] Schmid TE, Dollinger G, Beisker W, Hable V, Greubel C, Auer S, Mittag A, Tarnok A, Friedl AA, Molls M, Röper B. Differences in the kinetics of γ-H2AX fluorescence decay after exposure to low and high LET radiation. Int J Radiat Biol 2010, 86 (8): 682-691. 10.3109/09553001003734543 20569192

[33] Hegemann NS, Guckenberger M, Belka C, Ganswindt U, Manapov F, Li M (2014). Hypofractionated radiotherapy for prostate cancer. Radiother Oncol.

[34] Fukuda H, Hiratsuka J, Kobayashi T, Sakurai Y, Yoshino K, Karashima H, Turu K, Araki K, Mishima Y, Ichihashi M (2003). Boron neutron capture therapy (BNCT) for malignant melanoma with special reference to absorbed doses to the normal skin and tumor. Australas Phys Eng Sci Med.

[35] Andoh T, Fujimoto T, Sudo T, Fujita I, Imabori M, Moritake H, Sugimoto T, Sakuma Y, Takeuchi T, Kawabata S, Kirihata M, Akisue T, Yayama K, Kurosaka M, Miyatake S, Fukumori Y, Ichikawa H (2011). Boron neutron capture therapy for clear cell sarcoma (CCS): biodistribution study of p-borono-L-phenylalanine in CCS-bearing animal models. Appl Radiat Isot.

[36] Fujimoto T, Andoh T, Sudo T, Fujita I, Moritake H, Sugimoto T, Sakuma T, Akisue T, Kawabata S, Kirihata M, Suzuki M, Sakurai Y, Ono K, Fukumori Y, Kurosaka M, Ichikawa H (2013). Boron neutron capture therapy (BNCT) selectively destroys human clear cell sarcoma in mouse model. Appl Radiat Isot.

[37] Pickering DG, Stewart JS, Rampling R, Errington RD, Stamp G, Chia Y (1987). Fast neutron therapy for soft tissue sarcoma. Int J Radiat Oncol Biol Phys.

[38] Zhongtai M, Huaiguang L, Wenjiang S (1994). Fast neutron radiotherapy for osteosarcoma. Chinese J of Can Res.

[39] Cohen L, Hendrickson F, Mansell J, Kurup PD, Awshalom M, Rosemberg I, Haken RKT (1984). Response of sarcoma of bone and of soft tissue to neutron beam therapy. Int J Radiat Oncol Biol Phys.

[40] Schwartz DL, Einck J, Bellon J, Laramore GE (2001). Fast neutron radiotherapy for soft tissue and cartilagimous sarcoma at high risk for local recurrence. Int J Radiat Oncol Biol Phys.

[41] Glaholm J, Harmer C (1988). Soft-tissue sarcoma: neutrons versus photons for postoperative irradiation. Brit J Rad.

[42] Menendez PR, Roth BMC, Pereira MD, Casal MR, Gonzalez SJ, Feld DB, Santa Cruz GA, Kessler J, Longhino J, Blaumann H, Jimenez Rebagliati R, Calzetta Larrieu OA, Fernandez C, Nievas SI, Liberman SJ (2009). BNCT for skin melanoma in extremities: updated argentine clinical results. Appl Radiat Isot.

[43] Schwarz R, Bruland O, Cassoni A, Schomberg P, Bielack S. The role of radiotherapy in Oseosarcoma, in pediatric and adolescent Osteosarcoma. In: Jaffe N, Bruland OS, Bielack S, editors. Cancer treatment and research: Springer; Dordrecht Heidelberg 2009;152:147–164. 10.1007/978-1-4419-0284-9_7 20213389

[44] Machak GN, Tkachev SI, Solovyev YN (2003). Neoadjuvant chemotherapy and local radiotherapyfor high-grade osteosarcoma of the extremities. Mayo Clin Proc.

[45] Gaitan-Yanguas M (1981). A study of the response of osteogenic sarcoma and adjacent normal tissues to radiation. Int J Radiat Oncol Biol Phys.

[46] Mottard D, Grimer RJ, Abudu A, Carter SR, Tillman RM, Jeys L, Spooner D (2012). Biological reconstruction after excision irradiation and reimplantation of diaphyseal tibial tumours using an ipsilateral vascularised fibular graft. J Bone Joint Surg (Br).

[47] Calvo FA, Ortiz de Urbina D, Sierrasesumaga L (1991). Intraoperative radiotherapy in the multidisciplinary treatment of bone sarcomas in children and adolescents. Med Pediatr Oncol.

[48] Tallman MP, Williams JP, Eagleton MJ, Hernady E, Rubin P, Pomerantz RM (1999). Tolerance of normal rabbit femoral arteries to single high dose external beam irradiation. Cardiovasc Radiat Med.

[49] Kinsella TJ, De Luca AM, Barnes M, Anderson W, Terrill R, Sindelar WF (1991). Threshold dose for peripheral neuropathy following intraoperative radiotherapy (IORT) in a large animal model. Int J Radiat Oncol Biol Phys.

